# Magnetically Tunable Micro-Ring Resonators for Massive Magneto-Optical Modulation in Dense Wavelength Division Multiplexing Systems

**DOI:** 10.3390/s22218163

**Published:** 2022-10-25

**Authors:** Josino Villela S. Neto, William O. F. Carvalho, Jorge Ricardo Mejía-Salazar

**Affiliations:** National Institute of Telecommunications (Inatel), Santa Rita do Sapucaí 37540-000, MG, Brazil

**Keywords:** dense wavelength division multiplexing, magneto-optic modulator, micro-ring resonators, optical modulator, photonic integrated circuits

## Abstract

We demonstrate, numerically, a new concept for on-chip magneto-optical (MO) modulation in dense wavelength division multiplexing (DWDM) applications. Our idea uses materials and mechanisms that are compatible with current silicon-on-insulator fabrication and CMOS technologies for monolithic integration. The physics behind our idea stems in the exploitation of the enhanced MO activity of a micro-ring, made of cerium substituted yttrium iron garnet (Ce:YIG) material, to actively manipulate the resonance wavelengths of an adjacent micro-ring resonator (MRR) of silicon (Si). This active manipulation of the latter MO-MRR structure is used to modulate the optical signal traveling through a side-coupled Si bus waveguide. Moreover, by proper tailoring multiple MO-MRRs (side-coupled to the single Si bus waveguide) to match wavelength channels in DWDM across the entire C-band optical communications spectrum, we extend our proposal to massive and dynamic MO modulation in DWDM applications. Significantly, we noticed that the active MO shifting of the resonant wavelength (used for MO modulation here) can be used for improvements in the spectrum utilization efficiency in future elastic optical networks (EONs).

## 1. Introduction

Owing to the increasing popularity of video streaming, mobile and cloud computing services, data traffic in optical communications networks is experiencing explosive growth during the last few years. This trend seems to be unbroken, at least during the upcoming decade, due to sustained innovations in wireless communications and its seamless integration into existing fibre-optic infrastructures [1,2]. Hence, improvements in the spectrum utilization efficiency of optical transport networks are needed to cope with the associated challenges. Moreover, seamlessly integrated photonic and nano-electronic circuits are required for high-fidelity optical signal processing, which demands monolithic (i.e., on a single chip) integration of photonic circuits into CMOS (complementary metal-oxide semiconductor) technology [3,4].

Current approaches to meet the increasing capacity and bandwidth requirements (in optical communications networks) are mostly based on the use of wavelength division multiplexing (WDM) systems [5,6], where the optical transmission spectrum of a single optical fiber is divided into multiple channels of non-overlapping wavelengths. Importantly, each single communication channel can operate at whatever speed it desires by exploiting the complementary physics between electronic and photonic technologies [7]. This level of integrability (at the chip scale) is reached through the silicon-on-insulator (SOI) micro-ring resonators (MRRs), which are essential building blocks for channel add/drop filters [8,9,10], interleavers [11], and multiplexing/demultiplexing devices [12] in advanced optical communications networks. In particular, sharp optical resonances associated to MRRs are used to add/extract specific wavelengths without disturbing other channels in WDM. Furthermore, the CMOS compatibility of SOI technology enables high-density integration of photonic-to-electronic functions on a chip [3,4]. However, in spite of these advantages, the spectrum utilization efficiency of WDM is still limited by the fixed channel and grid spacing from conventional approaches.

On the other hand, to continue scaling optical communications networks, both in density and complexity, it is necessary to replace the conventional metallic intra- and inter-chip data links (limited by losses, bandwidth, and crosstalk) with their optical counterparts [13,14,15,16]. The workhorse of an optical interconnect is the optical modulator, i.e., the device used to load information onto the light beam [17,18,19,20]. According to the physical principle, modulators can be classified as electro-optical (EO) [21], thermo-optical (TO) [22] and, more recently, magneto-optical (MO) [23,24,25]. In EO modulation, for example, an external electric field is used for dynamic manipulation of the guided light intensity and phase [3,26]. Although recent EO modulation devices exploit the unique properties of advanced materials (e.g., graphene) to improve the energy and modulation performances [27,28,29], their operation speeds are still considered limited [30]. In the case of TO modulators, the features of the optical signal are manipulated by temperature changes (induced through the thermoelectric effect) [22]. However, this latter modulation mechanism results in vulnerable optical circuits (susceptible to thermal fluctuations) and additional complexity (in terms of design and components) for temperature stabilization [31]. The MO approach, in contrast, employs an external magnetic field for optical signal modulation. This last approach overcomes the limitations associated to EO and TO modulation mechanisms [32,33]. Nevertheless, current MO modulation approaches are based in the polarization conversion effect, via the MO Faraday effect, which can introduce unwanted noise and impair optical-to-electronic (electronic-to-optical) coupling.

In this work, we numerically demonstrate a new concept for MO modulation and/or dynamic WDM applications. MO activity is introduced using the cerium substituted yttrium iron garnet (Ce:YIG) material, which has recently been monolithically integrated (through both SOI and CMOS compatibility) into broadband optical isolators [34,35] and non-volatile MO switches [36]. Furthermore, a Si-based MRR, with an inner Ce:YIG MO disk, was recently used for dynamic add/dropping of modes between two coupled waveguides [37]. To develop our idea, we first use a single racetrack-like MO-MRR, side-coupled to a single Si-waveguide, to demonstrate a concept for amplitude-based MO modulation, i.e., without polarization conversion of the optical signal. The MO-MRR is built by two adjacent and concentric micro-rings. The external micro-ring is made of Si to provide the corresponding resonances on the guided modes, whereas the inner micro-ring made of Ce:YIG for MO modulation of the Si micro-ring resonances. Second, we use a linear array of MO-MRRs side-coupled to a single bus waveguide for massive modulation on a single chip. More specifically, we show that this last structure can be used for dynamically tunable dense WDM (DWDM) along the optical C-band (1530 nm up to 1565 nm), with a free spectral range (FSR) of 100 GHz. Finally, it is worth mentioning that the modulation mechanism shown here reaches extinction ratios (ERs) in the order of 46 dB which, as shown comparatively herein, are higher than all previous modulation approaches.

## 2. Theory and Modeling

**Figure 1:**
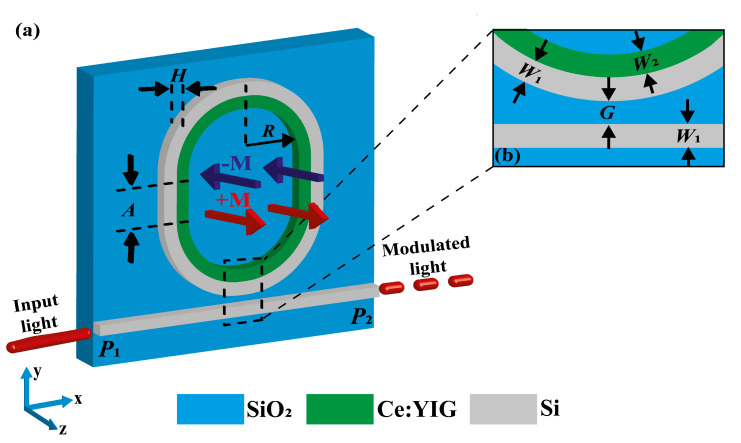
(**a**) Schematic 3D of the MO-MRR and (**b**) a zoom-in of the highlighted region in (**a**).

Figure 1a,b show a three-dimensional (3D) view and an upper view of the proposed design. The MO-MRR is formed by two adjacent and concentric micro-rings with a racetrack shape and side-coupled to a straight waveguide. The external MRR and the straight waveguide are both composed of Si, whilst the inner micro-ring is made of MO Ce:YIG material. The high-refractive-index contrast of Si provides waveguiding of the optical signals in the structures, whereas the Ce:YIG material is used for MO modulation of the resonant wavelengths in the external Si-MRR. The substrate is considered of silica (SiO_2_), which is compatible with SOI and CMOS technologies. The Si waveguide and MRR are considered with the same width $W_{1}$ and height *H*, whilst the inner Ce:YIG micro-ring has width $W_{2}$ and height *H*. The inner radius of the Ce:YIG micro-ring semicircular sections is denoted by *R* (see Figure 1), whilst the straight waveguide section has length *A*. The gap between the Si-MRR and the Si-waveguide is denoted by *G*. The system in Figure 1 can be monolithically integrated through the use of pulsed laser deposition (PLD) [34,38], nanolithography [39], or a proper combination of these and other methods for growing materials. Moreover, although magnetic fields can be applied at the chip-scale using integrated Si-compatible electromagnetic coils, as recently demonstrated [36], results in this work were obtained using the intrinsic magnetization of the CeYIG material (at magnetization saturation level).

The input signal in the system is applied through the port 1 ($P_{1}$) and propagates to the port 2 ($P_{2}$), as illustrated in Figure 1a. As it is well-known from the available literature [40], under the phase-matching condition, the guided optical mode in the Si-waveguide is resonantly coupled to the Si-MRR. This phase-matching condition states that resonances occur when the wavelength of the optical beam fits a whole number of times inside the optical length of the ring. Then, the system exhibits a set of resonances described by [40,41]

(1) $$\lambda_{\mathrm{res}}^{\pm }=\frac{n_{\mathrm{eff}}^{\pm }L}{p},$$

where $\lambda_{\mathrm{res}}^{\pm }$ is the resonance wavelength, $n_{\mathrm{eff}}^{\pm }$ is the effective refractive index of the guided mode, *L* is the optical path along the ring and p=1,2,3,... is an integer that defines the multiple of the wavelength that characterizes the resonance. Since we are considering MO activity in the proposed device, the superscript ± is used to indicate the magnetization (M) sense along the magnetized axis (*z*-axis in this case, as illustrated in Figure 1a), i.e., $\lambda_{\mathrm{res}}$ and $n_{\mathrm{eff}}$ depend on the magnetization sense in the structure.

MO effects can induce phase, polarization, and/or amplitude changes in the optical field depending on the magnetization configuration in the system [42]. In particular, MO activity only induces amplitude changes on the optical mode when the magnetization is parallel to the magnetic field component of light. Therefore, we restrict, here, our analysis to the case of guided modes with their magnetic field component along the *z*-axis, i.e., parallel to magnetization of Ce:YIG illustrated in Figure 1a. The anisotropic permittivity of Ce:YIG for this configuration is represented by the permittivity tensor

(2) $${\tilde{\varepsilon }}_{\mathrm{Ce}:\mathrm{YIG}}=\left(\begin{array}{ccc} \varepsilon_{\mathrm{Ce}:\mathrm{YIG}} & iB\varepsilon_{xy} & 0\\ -iB\varepsilon_{yx} & \varepsilon_{\mathrm{Ce}:\mathrm{YIG}} & 0\\ 0 & 0 & \varepsilon_{\mathrm{Ce}:\mathrm{YIG}} \end{array}\right),$$

where $\varepsilon_{\mathrm{Ce}:\mathrm{YIG}}$ represents the isotropic diagonal permittivity component of Ce:YIG, B=±1 is used for M pointing along the ±z direction, and $\varepsilon_{yx}=\varepsilon_{xy}$ are the anisotropic off-diagonal components. The values of $\varepsilon_{\mathrm{Ce}:\mathrm{YIG}}=5.098+i0.018$ and $\varepsilon_{yx}=\varepsilon_{xy}=8\times 10^{-3}$ are used from the experimental measurements in Ref. [43]. For simplicity, calculations are made within the magnetic saturation condition, which requires magnetic field amplitudes of ∼2 kOe [43]. On the other hand, the isotropic permittivities of Si and SiO${}_{2}$ are used from experimental values as $\varepsilon_{\mathrm{Si}}=12.08$ [44], $\varepsilon_{{\mathrm{SiO}}_{2}}=2.08$ [45], respectively. All permittivities are considered constant due to negligible changes ($10^{-3}$ for the diagonal and $10^{-4}$ for the off-diagonal components) within the working wavelength range.

We optimized the proposed device to work in the fundamental mode, considering that the magnetic field component of the guided wavelength ($H_{z}$) is along the *z*-axis. As the current lasers for WDM systems are mostly available to operate around λ=1550 nm [46], we used λ=1550.44 nm for the geometrical designs of the MO-MRR and straight waveguide. We start the optimization by looking for the lowest insertion loss (IL) on the straight waveguide, i.e., the highest transmittance level at $P_{2}$, in the non-resonant condition. Then, we optimized the gap between the straight waveguide and the MO-MRR by searching for the best wave coupling with the lowest IL in the system. Through the corresponding numerical results/analysis, we obtained $W_{1}=W_{2}=340$ nm, R=5.6μm, A=600 nm, $G_{1}=82$ nm, and H=525 nm for the geometrical parameters in Figure 1. We should remark here that, to avoid the use of large computational resources and time-consuming calculations, we carried out simulations of a 3D device by using two complementary 2D simulations. First, we calculate the mode effective index for a simplified longitudinal 2D structure (along the *xy*-plane) with optimized waveguides widths ($W_{1}$ and $W_{2}$). Then, using the effective index and widths information, in the second 2D simulation we calculate the waveguides cross-sections to find the corresponding heights. The input port, named $P_{1}$, was excited with 1 mW, whereas the output port, named $P_{2}$, was used to measure the transmittance (labeled as $T_{21}$), as illustrated. Numerical results were obtained using the finite element method (FEM) with the commercial software COMSOL multi-physics^®^ (Stockholm, Sweden). Calculations as function of the wavelength were swept using steps of 0.001 nm. The mesh was used with finer sizes of λ/10 around the waveguides and λ/5 in the farthest region from the waveguides, with a growth transition rate of 1.1. Moreover, scattering boundary conditions and absorbing perfect matched layers (PMLs) were used to avoid spurious numerical reflections.

## 3. Results and Discussion

**Figure 2:**
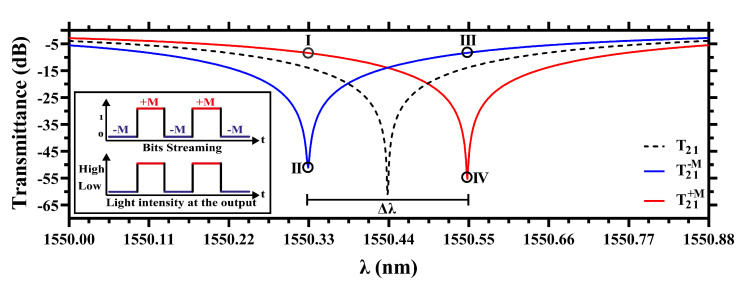
Transmittances in the MO-MRR for non-magnetized (black dashed curve) and magnetized to −M (blue solid curve) and +M (red solid curve). The points I and III symbolize a possible coding for bit 1, whilst the points II and IV may coding the bit 0, as depicted in the inset.

Let us start discussing the MO modulation mechanism. In this case, our attention is focused around the working wavelength λ=1550.44 nm, within a small wavelength range of the C-band (from 1550 nm to 1550.88 nm), where $T_{21}$ is calculated as a function of λ. Numerical results are comparatively shown for B=0 (i.e., the demagnetized system) and B=±1 in Figure 2. The strong resonance at 1550.44 nm (for B=0) is verified numerically with a very deep transmittance drop $T_{21}=-61.3$ dB. For B=1 (B=−1) we note that the transmittance deep suffers a resonance shift of 0.11 nm (−0.11 nm), with a relatively small change (<7 dB) in the transmittance deep. The symmetric displacement of resonance around the working wavelength, for B=±1, totalize a wavelength shift of Δλ=0.22 nm (in the magnetic saturation condition), as indicated in Figure 2. At λ=1550.33 nm (λ=1550.55 nm) the marks I and II (III and IV) indicate the wavelength where our design can work as a MO modulator. Indeed, considering two levels of output intensity, a non-return-to-zero (NRZ) encoded bitstream can be assumed using the magnetization sense along the *z*-axis, e.g., B=1 for bit 1 and B=−1 for bit 0, as depicted in the inset of Figure 2.

**Figure 3:**
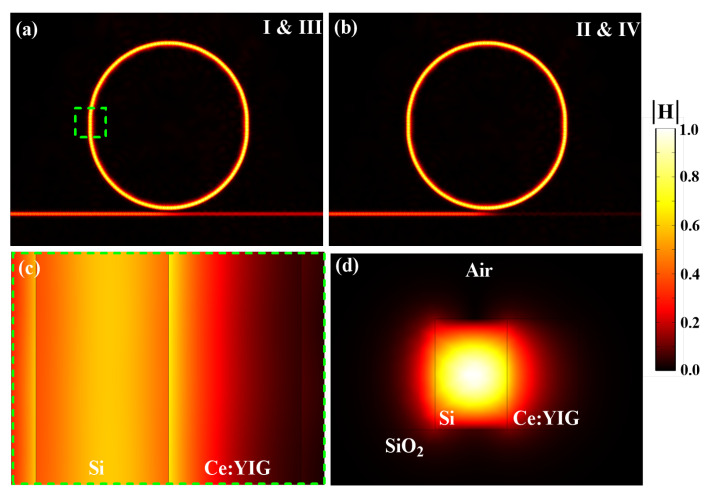
Normalized *H*-field in the MO-MRR. (**a**) The system in non-resonance condition (I & III) and (**b**) in resonance condition (II & IV). In (**c**), an inset from (**a**) at the straight MRR section, where one can see the guided wave and the evanescent field along the MO ring. (**d**) the cross-section view of the MO-MRR for TM mode.

The ER, defined as the difference between the IL and the resonance deep, is as important parameter to measure the modulation performance. Calculating the ER for the proposed structure working at λ=1550.33 nm (λ=1550.55 nm), indicated by points I & II (III & IV) in Figure 2, we obtain ER=42.6 dB (ER=46.7 dB). This result is remarkably high in comparison to other recent works, as it is shown in Table 1. Moreover, it can be also noted from the Table 1 that the radius of our proposal is relatively small (with a small footprint of 1723 μm^2^), enabling high density on-chip integration. The upper view of the magnetic field profiles associated to the modes I & III and II & IV are shown in Figure 3a,b, respectively. For visualization purposes, the region highlighted in Figure 3a is zoomed in Figure 3c. From this last figure we note that the guided mode in the MRR of Si is distributed inside the adjacent micro-ring of Ce:YIG, where stems the physical principle in this work (through the enhanced MO activity of Ce:YIG). A cross-section view of the magnetic field profile in the highlighted region is shown in Figure 3d, where the lower refractive index contrast at the Si-Ce:YIG interface (compared with the Si–air interface) explains the higher evanescent light penetration into the Ce:YIG material.

**Figure 4:**
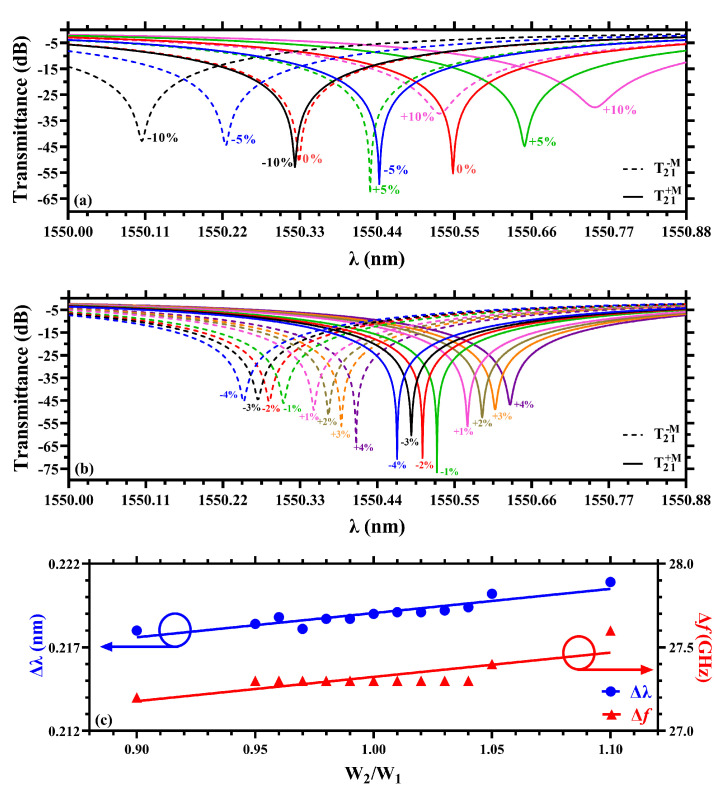
Resonances for ±M with small differences between the widths $W_{2}$ and $W_{1}$. (**a**) Variations with steps of ±5% from 0% to ±10% and (**b**) variation from −4 to +4% with steps of 1%. (**c**) A linear fitting of Δλ as function of $W_{2}/W_{1}$.

For practical implementations of our proposal, it becomes necessary for an analysis of the tolerance to fabrication errors. Although several nanolithography techniques have been developed for precise fabrication of MRRs [40,41], due to the high sensitivity of sharp resonances to the micro-ring radius and waveguide dimensions, the sensitivity of MO activity to small manufacturing errors deserves attention in this work. So far, the inner Ce:YIG MRR was optimized to have $W_{1}=W_{2}$, which, in turn, produce nearly the same transmittance dips for ±M, as seen in Figure 2, with a slight difference of ∼3.9 dB. Figure 4a shows calculations of $T_{21}$ for the system with $W_{2}=W_{1}\pm \delta W_{1}$, using δ=0,±5%,±10%, whereas results for δ=±1%,±2%,±3% and ±4% are shown in Figure 4b, where solid and dashed curves are used for + and −. These results indicate resonance shifts and increasing difference between the transmittance dips for ±M, except for δ=2% where a difference as small as 0.4 dB is obtained. Interestingly, a linear dependence of Δλ with the ratio $W_{2}/W_{1}$ was noticed, however, due to the negligible small slope m=0.01387, we can consider Δλ as constant, as illustrated by the blue curve in Figure 4c. Additionally, for sake of clarification, the resonance difference in the frequency domain (Δf) shows a constant behavior, as it can be observed. Therefore, fabrication errors should not be higher than $W_{2}=W_{1}+2\%W_{1}$ to guarantee almost the same level of MO activity for ±M.

**Figure 5:**
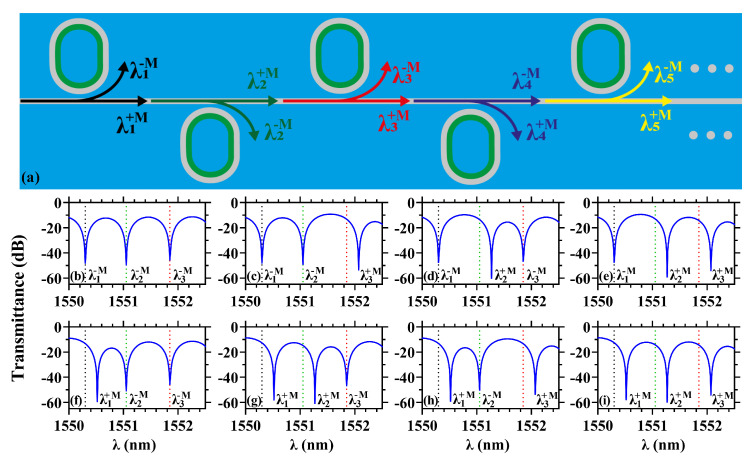
(**a**) Schematic representation of multiple MO-MRRs side-coupled to a single Si-waveguide for massive MO modulation in DWDM applications. (**b**–**i**) MO modulation in DWDM for different combinations of $\left(\lambda_{1}^{\pm M},\lambda_{2}^{\pm M},\lambda_{3}^{\pm M}\right)$.

On the other hand, our concept can be extended along the entire optical C-band (1530∼1565 nm), for massive MO modulation, by using a bus waveguide side-coupled with a linear arrange of MO-MRRs. A schematic representation of the system is shown in Figure 5a, where the straight waveguide and racetrack-like MO-MRRs are illustrated. Considering that on-chip integrated electromagnets can be manufactured with available fabrication techniques [36,50], we assume that the magnetization sense of each individual MO-MRR can be manipulated at will. This last application, therefore, enables a new concept for magnetically tunable DWDM applications (allowing for a better harnessing of the available optical spectrum), where different wavelength channels can be dynamically accessed/modulated through the use of locally applied magnetic fields. It is worth mentioning that we are working in the state of magnetization saturation for the Ce:YIG material, that is, magnetic fields in the system are of the order of 0.2 T, with MRRs far apart (to avoid any type of coupling). Therefore, after the system is magnetized, the external magnetic field can be turned off to prevent unwanted crosstalk or other effects that the external magnetic field may induce. A major issue to be avoided is cross-talk (CT) among adjacent optical channels, which, in DWDM, are separated by a fixed FSR value of 200 GHz (Δλ=1.6 nm), 100 GHz (Δλ=0.8 nm), 50 GHz (Δλ=0.4 nm), etc., following the International Telecommunication Union (ITU-T) [51]. After numerical analysis, we found that the minimum FSR allowed to avoid CT is 0.8 nm, i.e., our device can work in DWDM with an FSR≥100 GHz. Consequently, we fixed FSR=100 GHz and designed the corresponding MO-MRRs lengths to modulate each one of the 44 different optical channels in the C-band. The corresponding numerical results are shown in Figure 5b–i which, for the sake of presentation, were limited to the wavelength range from 1550 nm to 1552.5 nm. In these latter figures, we use three optical channels labeled $\lambda_{1}^{-M}=1550.3$ (black dotted-line), $\lambda_{2}^{-M}=1551.05$ (green dotted-line), and $\lambda_{3}^{-M}=1551.85$ (red dotted-line), with eight combinations of ±M, to show that each single optical channel can be modulated without interfering on the resonant behavior of the adjacent channels. Importantly, the active manipulation of individual resonant wavelength channels in Figure 5 can be used for future developments in elastic optical networks (EONs) which, in contrast to DWDM, can have a mixture of different FSR values (on an as-needed basis) to provide almost any line rate [5,6].

A guide for design of MO-MRRs is given in Table 2, where geometric changes used to provide the different resonances in Figure 5 are shown. In particular, it should be noted that only the straight section of the race-track shape, *A*, is altered to obtain all the different resonances compatible with the optical carriers in DWDM systems [51]. As noticed, the resonance wavelengths exhibit a linear increasing/decreasing with increasing/decreasing *A*, which indicates a very simple way to implement this concept. In particular, Figure 5 presents the MO-MRRs with $A_{1}=600$ nm, $A_{2}=613$ nm, and $A_{3}=626$ nm, which are spaced by ΔA=13 nm, leading to resonance shifts of around 0.8 nm (required to reach the FSR=100 GHz).

## 4. Conclusions

In summary, we numerically shown a concept for MO modulation based in the use of MO-MRRs. Our results indicate highly efficient modulation of a single wavelength channel (using a single MO-MRR) or multiple individual channels in DWDM applications (using a set of MO-MRRs). Significantly, we considered materials, geometries, and components, that can be monolithically integrated with currently available manufacturing methods. Although we fixed FSR=100 GHz, we noticed from the active resonance shifting that future implementations in EONs are feasible with our idea. In particular, from results for massive MO modulation in DWDM, it can be seen that the active modulation of a specific wavelength channel does not disturb/overlap the other channels in the system. Remarkably, we reached a MO modulation depth of ER=46.7 dB which, as comparatively shown, is at least 1.7 times higher than the best result from previous literature.

## Figures and Tables

**Table 1:** Features comparison among the optical effects in MRR-based modulators.

Ref.	Waveguide	Substrate	Materials for Tunability	Effect	Ring Radius	IL	ER	Δλ
[47]	Si	Si (CMOS)	p-Si and n-Si	EO	12 μm	1 dB	2.4 dB	0.06 nm
[28]	Si	SiO${}_{2}$	Graphene	EO	3 μm	1.54 dB	10.2 dB	6 nm
[27]	Si${}_{3}$N${}_{4}$	SiO${}_{2}$	Graphene	EO	40 μm	6 dB	28 dB	0.2 nm
[30]	LiNbO${}_{3}$ and Si	SiO${}_{2}$	aluminum	EO	15 μm	4.3 dB	5.6 dB	0.066 nm
[48]	Si	Si (CMOS)	p-Si and n-Si	EO	8 μm	3–6 dB	7.78 dB	0.02 nm
[22]	Si	SiO${}_{2}$	p-Si and n-Si	TO	10 μm	4–14 dB	20 dB	0.05 nm
[49]	Si	Si (CMOS)	TiN	TO	8 μm	13 dB	25 dB	0.6 nm
[24]	Si${}_{3}$N${}_{4}$	SiO${}_{2}$	Cobalt nanoparticles	MO	10 μm	0.6 dB	4.75 dB	-
This work	Si	SiO${}_{2}$	Ce:YIG	MO	5.6 μm	5 dB	46.7 dB	0.22 nm

**Table 2:** Length *A* for each optical channel modulation spaced, for 100 GHz DWDM systems.

*A* (nm)	$\lambda_{\mathrm{res}}^{-}$ (nm)	$\lambda_{\mathrm{res}}^{B=0}$ (nm)	$\lambda_{\mathrm{res}}^{+}$ (nm)	Δλ (nm)	IL (dB)	ER (dB)
561	1547.91	1548.02	1548.13	0.22	10.95	38.85
574	1548.73	1548.84	1548.95	0.22	10.76	43.31
587	1549.54	1549.64	1549.76	0.22	11.26	42.44
600	1550.33	1550.44	1550.55	0.22	12.34	46.70
613	1551.10	1551.21	1551.32	0.22	11.60	35.60
626	1551.87	1551.98	1552.09	0.22	11.37	33.47
639	1552.75	1552.86	1552.97	0.22	11.83	41.19
652	1553.54	1553.65	1553.76	0.22	11.81	35.25
665	1554.33	1554.44	1554.55	0.22	11.25	29.42
678	1555.16	1555.28	1555.39	0.23	10.92	24.76

## Data Availability

Data underlying the results presented in this paper are not publicly available at this time but may be obtained from the authors upon reasonable request.
